# Essential thrombocythemia: nutritional management in weight loss and malnutrition

**DOI:** 10.11604/pamj.2024.47.93.32594

**Published:** 2024-02-28

**Authors:** Isabela de Souza da Costa Brum, Julia Goncalves, Mariana Zanchetta, Bruna Xerém, Renata Lanziani, Marcia Haiut, Masato Hada, Alexandre Gustavo Apa, Karen Cordovil

**Affiliations:** 1 Institute of Hematology Arthur Siqueira Cavalcanti (HEMORIO), Rio de Janeiro, RJ, Brazil,; 2 Fluminense Federal University, Niteroi, Rio de Janeiro, RJ, Brazil,; 3 Oswaldo Cruz Foundation (FIOCRUZ), Rio de Janeiro, RJ, Brazil,; 4 Hada Clinic, Numazu, Japan,; 5 National Cancer Institute (INCA), Rio de Janeiro, RJ, Brazil

**Keywords:** Essential thrombocythemia, body mass loss, cytokine

## Abstract

Essential thrombocythemia is the category of myeloproliferative syndromes, generally characterized by a group of clonal stem cell diseases that present a disturbance in the growth of one or more sets of hematopoietic cells. All long clinical treatment, patients may experience gastrointestinal disorders and other metabolic processes that can lead to weight loss and malnutrition. Cytokine is involved in the control of appetite, digestive, and metabolic processes in the body, it can be assumed that increased stimulation could impair the control of these processes leading to loss of body mass. Effective and systematic nutritional intervention is required to ensure patient compliance with treatment and improved nutritional status.

## Commentary

Myeloproliferative syndromes (MS) are a group of clonal stem cell diseases that have a disorder in the growth of one or more series of hematopoietic cells, leading to peripheral blood leukocytosis and increased erythrocyte mass or thrombocytosis, and MS is known to be divided into chronic myeloid leukemia (CML), polycythemia vera (PV), and essential thrombocythemia (ET) [1]. Essential thrombocythemia arises due to a clonal cell disorder and is made up of three hematopoietic cell lines, mainly the megakaryocytic line, responsible for platelet production and differentiation, where there is excessive proliferation leading to persistent thrombocytosis and increased red blood cell mass [1]. The estimated annual incidence in the United States of America is 2.5 cases per 100,000 habitants [1]. In Europe, the estimated incidence of ET ranges from 0.38-1.7 per 100,000 per year [2]. In South Africa, there is no data on the epidemiology of ET incidence in the National Cancer Registry [3]. In a study in Asia that covered the Middle East, Turkey, and Algeria, it was found that among 884 patients with myeloproliferative disease, the prevalence of ET was 42.2% [4]. “In Latin America, there is no epidemiological data from large cohort studies to provide basic treatment models [5]. The mechanism for the occurrence of this event is not fully understood. However, there are acquired genetic defects that may influence the etiology, such as the Janus kinase 2 gene mutation (JAK2V617F), which affects 35 to 70% of people with TE [6]. The JAK2 protein is a tyrosine kinase responsible for the phosphorylation of the transcriptional activating signal transducer molecule (STAT) and is a trigger of the cell cycle, serving as an intermediary between membrane receptors and signaling molecules [6]. The mutation is a one-off with thiamine-substituted guanine in exon 14 of the JAK2 gene, leading to the replacement of valine by phenylalanine at position 617 of the encoded protein [6]. Clinical manifestations are quite variable, about 35% of cases are asymptomatic and accidentally discover ET. However, clinical manifestations such as bone pain, night sweats, early satiety, dizziness, headache, acrocyanosis, erythromelalgia, hemorrhagic and thrombotic phenomena may occur [6]. The World Health Organization has established some criteria for the diagnosis of ET that should be present: platelet count greater than 450 x 109/L; bone marrow biopsy with megakaryocytic hyperplasia, presenting large and mature megakaryocyte groups with hyper lobulated nuclei; exclusion of other myeloproliferative diseases (PV, myelofibrosis and CML) and myelodysplastic syndrome; JAK2 positive or MPL positive mutation; absence of secondary causes of thrombocytosis; the presence of splenomegaly on physical examination or abdominal ultrasound, and the therapeutic approach in ET focuses on reducing the number of platelets and preventing thrombotic and hemorrhagic complications [1].

The most commonly used drug for treatment has been Hydroxyurea because of its cytoreduction purpose by inhibiting DNA synthesis of blocking the enzyme of ribonucleoside [7]. As already shown in studies, the presence of the JAK2 mutation may interfere with the response to Hydroxyurea by presenting more sensitivity to treatment, leading to a significant decrease in platelet count [7]. The use of this drug may lead to side effects: myelosuppression and neutropenia that require close monitoring during the first months and may even be a reason for stopping treatment; In addition to headache, dizziness, disorientation, hyperpigmentation, and gastrointestinal effects are also common: stomatitis, anorexia, constipation or diarrhea [7]. Constant weight loss in these patients may be due to the disease itself which has as one of its side effects weight loss [7,8]. The mechanism of disease is not fully understood, but the mutation of the JAK2 gene, which is one of the hematopoiesis signalers, causes loss of self-inhibitory control of JAK2, giving increased responsiveness to cytokines and independent signaling cytokines, leading to the activation of multiple signaling cascades [1,7,8]. Considering that cytokine is involved in the control of appetite, digestive and metabolic processes in the body, it can be assumed that increased stimulation could impair the control of these processes leading to loss of body mass [1,7,8]. Side effects of the main drug used in ET may influence body mass and these symptoms would negatively affect patients' quality of life and productivity [7,8]. Other factors that may be correlated with weight loss in patients with ET are: physiological factors that are related to loss of taste, sight, and smell; psychological factors that are related to environmental change and isolation; pathological factors are related to medical causes and medication use [8]. One consequence of loss of body mass when not corrected would be the onset of malnutrition, and more specifically in the elderly, sarcopenia [8-10]. Nutritional interventions should be performed according to the predominant clinical or semiological symptoms in a patient [9]. Generally, these symptoms are early satiety, neutropenia, anorexia, diarrhea, or constipation [10]. The objective of nutritional intervention for early satiety in this symptomatology is increased fractionation and reduced meal volume, evolving according to patient acceptance [10]. Calories should be concentrated in small volumes to ensure adequate caloric intake [9,10]. Also, the patients would avoid drinking drinks during the meal, very fatty preparations, and drinking carbonated drinks [8-10]. In the case of neutropenia, raw fruits and vegetables should be sanitized with sanitizers, as well as filtered, boiled, or mineral drinking water of good origin [10].

Give preference to cooked foods (fruits, vegetables, vegetables), with grains and oilseeds only being cooked [10]. The group of milk and pasteurized derivatives only, not fermented (yogurt and milk). Already the group of meat and eggs should be well cooked [8-10]. Processed foods must be in individual packages and within their shelf life. Sprouts and probiotics should not be used [10]. The nutritional intervention of anorexia should be performed mainly through patient awareness of the importance of eating, despite the lack of appetite. The strategies used are increased fractionation and reduced volume, concentrating calories and protein in small volumes; stimulation to eat the main meals (lunch and dinner) at the time the patient feels most hungry, and always having snacks ready to snack [10]. If the patient has diarrhea, the strategy of increasing fractionation and reducing meal volume should be used [10]. The healthcare professional should evaluate the need for lactose, sucrose, and gluten restriction and consider the use of prebiotics, probiotics, or symbiotics [10]. Hydration is important at this stage due to excess fluid loss, so replacement should be at least 3 liters [9,10]. The patient should be advised to avoid flatulent, hyperosmolar, and fatty foods, decrease the amount of insoluble fiber and increase or adjust the amount of soluble fiber and to drink isotonic drinks between meals to replace the losses. In patients with constipation, fractionation is normal but intervals between meals should be regular. Ingestion of fiber-rich foods with laxative characteristics, normal fluid intake, and exercise should be advised, as appropriate to the patient. The previous history should be collected carefully and should always be observed, as it traces the patient's path to seeking care. Also, one should collect family and social history [9]. To observe and consider the laboratory tests that were used for medical therapy for thrombocythemia.

If the patient is undergoing an important task, the physical and body staff should receive body mass restraints and perform body mass index (BMI) until ET confirmation. After this initial collection, it is advised that the service be performed with a nutritional and anthropometric assessment. Nutritional assessment should include food allergies and intolerances, presence of pica, intestinal functioning, water intake, the process of signs and symptoms related to nutritional nutrition or digestive tract (eg pigmented nails, dry skin, anorexia, motion sickness, etc.), food diary or 24-hour food recall [9]. An anthropometric evaluation should be performed in all consultations and deviations: body mass, height, skinfold, and circumferences [9]. To finalize a consultation, a semantic dietary control should be individually individualized, based on the main complaints and to encourage improvements in the quality of food intake. If the daily intake is not reached, the nutritional may use dietary supplements, as these can help both body mass gain and the reduction of some symptoms such as anorexia. The desire to study this topic was born from the experience of the nutrition team with some ET patients who arrived referred for nutritional monitoring and were losing weight. From this, the theme was searched for in the scientific literature, however, it was discovered that there it is a gap in the international literature. Even so, based on the hematological physiology and pathophysiology of ET, the authors established the practical and theoretical knowledge of clinical nutrition, considering the metabolic alterations of malnutrition, already well known in the international literature. We believe that this article can bring important new information that can help not only patients but all health professionals who work in the area of hematology. Our wish is that from this publication health professionals will pay more attention to the nutritional aspects of these patients, better monitoring their weight loss. In addition, we want scientists to read our article and conduct research that about this theme. Therefore, our greatest desire is to cooperate to advance policies and actions that prioritize health and nutrition care for all patients with hematological disease, including ET in Africa.

## Conclusion

As discussed, this article concludes that the action of cytokine and the side effects caused by treatment in ET possibly would cause loss of body mass in patients. An effective and systematic nutritional intervention is necessary to ensure patient compliance with treatment and improvement of nutritional status. The scarcity of studies relating to this topic in question reveals the great need to deepen the knowledge about the nutritional aspects of ET in the world and Africa.
